# Patterns of food preparation in children and adult diets and their associations with demographic and socio-economic characteristics, health and nutritional status, physical activity, and diet quality

**DOI:** 10.1017/jns.2024.87

**Published:** 2025-01-23

**Authors:** Mariana Correia Castro Rei, Daniela Macedo Correia, Duarte Paulo Martins Torres, Carla Maria Moura Lopes, Ana Isabel Almeida Costa, Sara Simões Pereira Rodrigues

**Affiliations:** 1 Faculdade de Ciências da Nutrição e Alimentação, Universidade do Porto, Porto, Portugal; 2 EPIUnit - Instituto de Saúde Pública, Universidade do Porto, Porto, Portugal; 3 Laboratório para a Investigação Integrativa e Translacional em Saúde Populacional (ITR), Universidade do Porto, Porto, Portugal; 4 Departamento de Ciências da Saúde Pública e Forenses, e Educação Médica, Faculdade de Medicina, Universidade do Porto, Porto, Portugal; 5 Católica Lisbon School of Business & Economics, Universidade Católica Portuguesa, Lisboa, Portugal

**Keywords:** Cross-sectional, Home cooking, Diet quality, Food preparation patterns, Public health factors

## Abstract

This cross-sectional study aimed to identify patterns of food preparation and examine their demographic and socio-economic drivers, along with impacts on health and nutritional status, physical activity, and diet quality. Dietary data from a national-representative sample (n = 5005, 3–84 years) of the Portuguese National Food, Nutrition, and Physical Activity Survey (IAN-AF 2015/16) were classified by preparation locations (at or away from home) and analysed via hierarchical clustering. Logistic regression models were used to examine associations between demographic and socio-economic factors and food preparation patterns and between these patterns and health and nutritional status, physical activity, and diet quality. The most common food preparation pattern (followed by 45.4% of participants) represented the highest intake of foods prepared by away-from-home establishments. Adolescents (vs. children, OR = 0.29, 95%CI = 0.17, 0.49) and older adults (vs. adults, OR = 0.37, 95%CI = 0.26, 0.53) had lower odds of following this pattern, whereas adult men (vs. women, OR = 4.20, 95%CI = 3.17, 5.57) had higher odds. Higher education, higher household income, and having children/adolescents in the household also increased the odds of eating foods prepared away from home, whereas living in rural areas or in food-insecure households decreased the odds. Noticeably, adults consuming more foods prepared away from home had lower odds of being overweight or obese (OR = 0.74, 95%CI = 0.56, 0.97), but higher odds of sedentarism (OR = 1.45, 95%CI = 1.08, 1.96) and poor diet (OR = 3.01, 95%CI = 2.08, 4.34) compared to those consuming more foods prepared at home by themselves. Dietary patterns marked by high away-from-home food preparation prevail. While these correlated with higher socio-economic status, sedentarism, and poorer diet — relatively to patterns with greater reliance on homecooked food — they were not linked to higher odds of obesity.

## Introduction

Chronic, diet-related noncommunicable diseases (NCDs) such as obesity, type 2 diabetes, hypertension, and several types of cancer are increasing worldwide, reflecting the vast and growing international burden of disease in this century, accounting for a significant proportion of global deaths and years of life lost.^(1–3)^ At the same time, the consumption of foods prepared away from home (in restaurants, takeaway and home delivery establishments, cafeterias and snack bars, or as ready-to-eat retail products, for instance) is also rising.^(4–6)^ On the contrary, the time spent on preparing food at home is decreasing, particularly in high-income countries,^(4–6)^ mainly due to time scarcity^(7,8)^. Changes in lifestyle associated with women’s expanded role in the paid workforce and an ever-increasing away-from-home food offer are likely driving both trends.^(9–11)^ The reconfiguration of cooking knowledge and skills and the social transformation of eating practices are equally noticeable, with the chores of the home kitchen being gradually replaced by the chores of shopping or driving for the next prepared meal.^(12,13)^ In parallel, away-from-home food consumption has been identified as a risk factor for higher total energy and fat consumption and lower micronutrient intake,^(14)^ as well as for becoming overweight.^(15)^ Altogether, this highlights the role that cooking and eating more at home, at the expense of less away-from-home food consumption, could play in promoting better diet quality and health status.^(4,16,17)^

Most national food consumption surveys do not record or record but do not prioritise the analysis of data on how the foods reportedly eaten are procured, prepared, and/or served or who was mainly in charge of these activities, except to support more accurate assessments of food and nutrient intake.^(18,19)^ A shortage of validated methods for classifying food intakes according to patterns of preparation compounds the problem further.^(20,21)^ Related studies of the prevalence of different patterns of food preparation at the population level — as well as of their demographic and socio-economic drivers and potential impacts on nutrition and health status — are therefore scarce, being furthermore geographically limited and predominantly focused on adults.^(6,22–30)^ Further research on patterns of food preparation and associated factors in Europe is restricted to cohort studies^(31–34)^ or analyses of national Time Use Survey data,^(5,35)^ with the notable exception of work using data from the North/South Ireland Food Consumption Survey 1997/99 to investigate associations between food preparation location and nutrient intakes in both adults and children.^(36–38)^ Patterns of food preparation and their correlates have not yet been the object of extensive research in Portugal, apart from work reporting on the time adults dedicated to food preparation at home in a nationally representative sample in 2015,^(39)^ and a regional cohort study providing data on adult food intake location between 1999 and 2003.^(40–42)^

Overall, these prior studies have shown that factors such as age, sex, and socio-economic status significantly influence eating habits, with notable differences in the frequency of consuming meals out and takeaway meals and in time spent on home food preparation.^(15,22,24,32,33,35,39,40,42)^ They also have shown distinct national patterns in eating practices, with variations in nutrient intakes and diet quality depending on whether food is prepared at home or outside.^(15,23,24,27–30,34,36–38,40–42)^ Therefore, based on the limited research on patterns of food preparation places, this cross-sectional study aims to provide a comprehensive overview of these patterns across the entire Portuguese population. Using data from the most recent National Food, Nutrition, and Physical Activity Survey (IAN-AF 2015/16), our study focuses on identifying patterns of food preparation in both children and adult diets. By uncovering demographic and socio-economic characteristics associated with these patterns, this study aims to better inform targeted public health strategies that promote healthier eating habits across various food preparation settings. Additionally, it is intended to explore the associations between patterns of food preparation and health and nutritional status, physical activity, and diet quality, offering valuable insights for public health policy and interventions.

## Methods

### Population and study sample

Participants in the IAN-AF 2015/16 (n = 6653) were randomly selected from the National Health Registry by multistage sampling and comprised a representative sample of the general (non-institutionalised) resident Portuguese population aged between 3 months and 84 years.^(43)^ This study analyses data from a sub-sample of survey participants aged between 3 years and 84 years, who completed two dietary intake assessments (n = 5005). Those who declined participation and completed a refusal questionnaire were generally older and less educated than participants, though differences in dietary consumption were minimal.^(43)^

### Dietary intake and diet quality

Dietary intake data were collected by trained researchers with a background in nutrition or dietetics, using an electronic platform (You eAT&Move) and following European Food Safety Authority (EFSA) recommendations for dietary assessment.^(43)^ Interviews 1–2 weeks apart were conducted over 12 months (from October 2015 to September 2016), distributed over the four seasons, and included all days of the week (randomly selected).^(43)^ Food consumption data were collected by two non-consecutive days of 24-h food diaries for children (<10 years) and two non-consecutive 24-h recalls for other age groups.^(43)^ Food photos for portion size estimation and automatic intake conversion using databases with the nutritional composition of foods and recipes were applied.^(43)^ For children, 24-h food diaries were followed by a face-to-face interview with the main caregiver, to gather details related to food description and quantification.^(43)^ For adolescents aged 10–14 years, 24-h recalls were administered with the presence of one of the caregivers; for adolescents aged 15–17 years, 24-h recalls were administered autonomously.^(43)^

Dietary intake data were used to assess diet quality by calculating the Mediterranean Adequacy Index (MAI).^(44)^ The higher the MAI, the closer a diet is to the Healthy Reference National Mediterranean Diet, a healthful diet in which food patterns are inversely correlated with the prevalence of risk factors for NCDs. MAI terciles were therefore used to classify the quality of participants’ diet as low, medium, or high.

### Demographic and socio-economic characteristics

Participants’ sex, date of birth, and parish of residence were drawn from the National Health Registry. Participants were grouped, according to age at date of first interview, into children (3–9 years), adolescents (10–17 years), adults (18–64 years), or older adults (65–84 years), and their parish of residence was classified as Predominantly Urban, Medium Urban, or Predominantly Rural according to the Portuguese National Institute of Statistics’ Classification of Urban Areas.^(45)^

Marital status, highest education completed, people living regularly in the household, and household monthly net income class were reported by adults and older adults only, during the first interview.^(43)^ Marital status was categorised as Not Married (single, widow/er, or divorced) or Married/Cohabiting, education as No Education/Primary Education, Secondary Education, or Tertiary Education, and household composition as Households without Children or Adolescents and Households with Children or Adolescents. Response options for household income class ranged from less than €485 to more than €4 365; answers were aggregated into three categories according to their dispersion: ≤ €970, €971–1 940, and >€1 940. For children and adolescents, the highest education level completed by their parents was reported and categorised similarly to that of adult and older adult participants.

A culturally adapted household food security questionnaire^(43,46)^ was administered to adults and older adults at the end of the second interview, and the results were used to classify the food insecurity status of their households as Food Security or Food Insecurity (mild and severe).

### Health and nutritional status and physical activity

Data on indicators of health and nutritional status were collected for all participants. The prevalence of any disease requiring regular medical care (no/yes) was collected using a pre-defined list of NCDs.^(43)^ For participants aged 3–17 years, the list included asthma, diabetes, gastrointestinal disease, or other diseases. For participants aged 18 years and older, the list included heart disease, stroke, cancer, type 1 diabetes, type 2 diabetes, high blood pressure, dyslipidaemia, gastrointestinal disease, depression, or other diseases. Body weight and height measurements were performed by trained researchers and participants classified into three classes of BMI — normal/underweight, overweight, or obesity — according to WHO standards.^(43,47)^

Physical activity data were collected for all participants aged 15 years or above using the short version of the International Physical Activity Questionnaire (IPAQ).^(43,48)^ The present study analyses data on physical activity from adult and older adult participants only, with these being classified as Inactive, Minimally Active, or Active accordingly to the IPAQ guidelines for data processing and analysis.^(49)^

### Classes of food preparation

**Figure 1.**
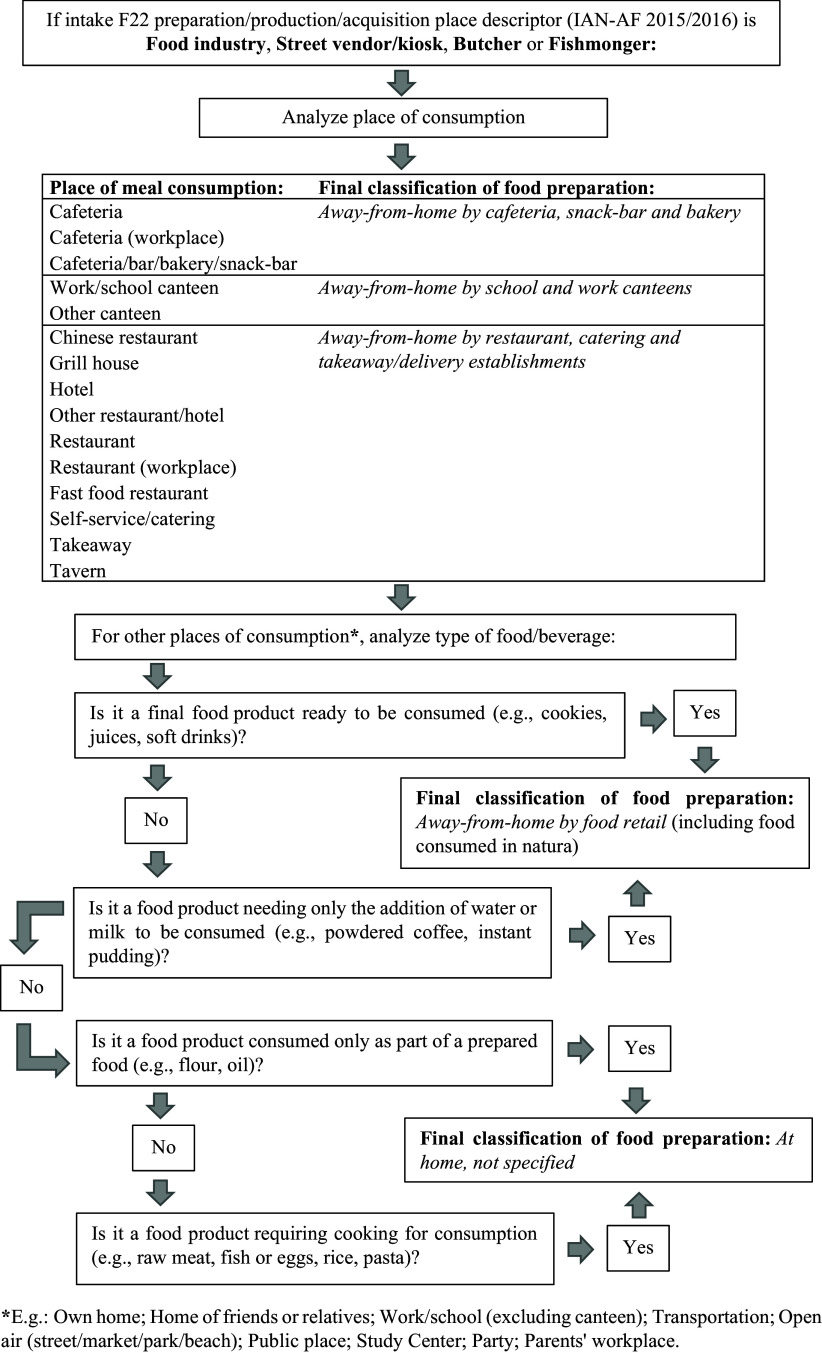
Decision diagram aiding in the classification of foods or beverages intakes of IAN-AF 2015/16 participants according to the classes of food preparation.

The eAT24 module^(50)^ of the IAN-AF data collection platform enabled interviewers to apply the EFSA FoodEx2 classification system^(51,52)^ to classify reported food intakes into three facets — foods, composite dishes/recipes, or food supplements — and corresponding descriptors, as applicable. A category of descriptors indicating the immediate food source (F22) or composite dish/recipe source (RF22) of a registered intake — titled Preparation/Production/Acquisition Place — was employed in the present study to reclassify intakes according to preparation locations. Table 1 and Figure 1 depict the steps and outcomes of this reclassification process, the development of which was informed by previous similar studies.^(20,24)^ Registered food intakes were reclassified by the first author and an expert reviewer independently, and resulting discrepancies were solved by a senior author.

**Table 1.** Classification of foods or beverages intakes of IAN-AF 2015/16 participants according to the classes of food preparation

Preparation/production/ acquisition place descriptor (IAN-AF 2015/16)	Final classification of food preparation
**Facets RF22 and F22**	
Undefined food production	*Undefined place*
Other	
At home (by him/herself)	*At home by him/herself*
At home (by friends)	*At home by relatives or friends*
At home (by relatives)	
Cafeteria/snack-bar	*Away from home by cafeteria, snack-bar and bakery*
Bakery	
Restaurant	*Away from home by restaurant, catering, and takeaway/delivery establishments*
Fast-food restaurant	
Catering	
Supermarket (ready-to-eat meals)	
Foodservice establishment (including takeaway or delivery)	
Work canteen/school canteen/university canteen	*Away from home by school and work canteens*
Vending machines	*Away from home by food retail* (including food consumed *in natura*)
Away from home, not specified	*Away from home, not specified*
**Facet RF22**	
Street vendor/kiosk	*Away from home by food retail* (including food consumed *in natura*)
Food industry (frozen or chilled pre-cooked meals)	
**Facet F22**	
Street vendor/kiosk (items purchased from a street vendor who prepared them, when applicable)	Dependent on outcome of decision diagram (Figure 1)
Food industry (items purchased at a supermarket, including those needing further preparation)	
Butcher	
Fishmonger	

### Statistical analysis

Mean daily intakes (%) of foods and beverages were calculated from the average food consumption (grams of edible weight) of each meal component recorded in the dietary assessments, for each class of food preparation considered. Values obtained for the class *Away from home, not specified* were negligible and hence excluded from further analysis. The remainder underwent hierarchical cluster analysis to uncover patterns of food preparation, using the Ward’s minimum variance method for agglomerating dissimilarities.^(53)^ The resulting cluster tree was trimmed from three to seven clusters, and a final four-cluster solution was achieved based on Dunn index values.^(53)^

The distribution of the patterns of food preparation (%) uncovered by cluster analysis was cross tabulated with the distributions (%) of the demographic and socio-economic characteristics of interest (sex, age group, degree of urbanisation of residence area, completed education, marital status, household composition, household monthly net income, household food insecurity status), health and nutritional status (NCDs, BMI), level of physical activity (IPAQ), and diet quality (MAI). The intakes of children and adolescents corresponding to a pattern of food preparation *At home by themselves* were negligible (<0.5%) and were therefore concatenated with the intakes corresponding to a pattern of food preparation *At home by relatives or friends*, to form an overall pattern of food preparation *At home*. In view of this, the number of food preparation patterns identified for adults/older adults and children/adolescents differed, leading to the performance of subsequent analyses independently for these two groups of participants.

Associations between demographic and socio-economic characteristics and patterns of food preparation were analysed by estimating logistic regression models, to obtain crude and adjusted odds ratios (OR and respective 95% confidence intervals (95%CI). The patterns of food preparation *At home by themselves* and *At home* were the reference categories in the case of adults/older adults and children/adolescents, respectively. Covariates were selected based on prior literature, theoretical considerations, and their potential to confound the relationships under study. Final models were adjusted for sex, age group, degree of urbanisation of residence area, or education, as applicable.

Logistic regression models were equally used to study the association between patterns of food preparation and the prevalence of NCDs (no vs. yes), nutritional status (normal/underweight vs. overweight and obesity), level of physical activity (active and minimally active vs inactive), and MAI (medium and high vs. low). Crude and adjusted odds ratios (OR) and corresponding 95% confidence intervals (95%CI) were also obtained, and the final models adjusted for total energy intake, sex, age group, degree of urbanisation of residence area, or education, as applicable.

All statistics were performed by the R Software version 3.6.3,^(54)^ and the sample was weighted for the Portuguese population distribution, using library ‘survey’.^(55)^ A significance level of α = 0.05 was considered in all analyses.

### Ethics

Ethical approval was obtained from the National Commission for Data Protection, the Ethical Committee of the Institute of Public Health of the University of Porto, and the Ethical Commissions of each of the Regional Administration of Health. All participants were also asked to provide their written informed consent for participation according to the Ethical Principles for Medical Research involving human subjects expressed in the Declaration of Helsinki and the national legislation. Written agreements from the legal representative were required for children and adolescents below 18 years, and adolescents were also asked to sign the consent form together with their legal representative.

## Results

### Dietary intake per class of food preparation

The foods and beverages consumed daily by Portuguese residents are predominantly prepared (or acquired, in the case of those consumed *in natura*) away from home, namely, at food retail establishments (45.8%, 95%CI = 44.8, 46.9%). Another important share of their diets (18.6%, 95%CI = 17.6, 19.5%) is prepared at home, but not by themselves. Self-prepared foods and beverages at home represent only slightly over 10% of mean daily intakes (12.2%, 95%CI = 11.2, 13.3%); all other food preparation classes represent less than 9%. Table S1 in Supplementary Materials shows the distribution of mean daily intakes (%) per class of food preparation.

### Patterns of food preparation

Cluster analysis identified four distinct patterns of food preparation among Portuguese residents (Table 2). The most prevalent pattern (followed by 45.4% of the participants) is characterised by the highest proportions of intakes of foods and beverages prepared by restaurants (13.9%), canteens (10.8%), and other foodservice establishments (9.6%) and is hence labelled *By restaurants, canteens, and other away-from-home establishments*. Specifically, about a third of the diet of individuals in this pattern is prepared away from home by foodservice operators. The second most prevalent (followed by 26.3% of the participants) exhibits the highest proportion — nearly two-thirds — of intakes of foods and beverages prepared by or acquired from food retail operators (including those consumed *in natura*) (65.3%); it is therefore named *By food retail*. The second least prevalent pattern (followed by 15.0% of the participants) distinguishes itself by having the highest proportion of intakes from foods and beverages prepared at home by individuals (38.0%) and is hence labelled *At home by themselves*. The least prevalent one (followed by 13.3% of the participants) displays the highest proportion of intakes from foods and beverages prepared at home by relatives or friends of individuals (44.7%); it is thus named *At home by relatives or friends*.

**Table 2.**
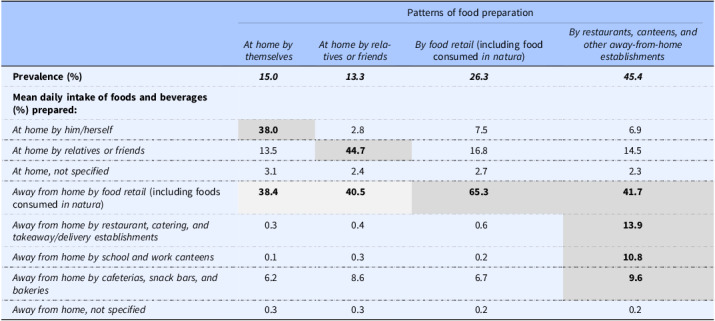
Patterns of food preparation identified for the Portuguese population using hierarchical cluster analysis (n = 5005; 3–84 years)

Tables S2 and S3 in Supplementary Materials show the distributions of the patterns of food preparation by the classes of demographic and socio-economic characteristics, health and nutritional status, level of physical activity, and diet quality considered.

### Demographic and socio-economic characteristics

#### Children and adolescents

Table 3 depicts the associations between demographic and socio-economic variables and patterns of food preparation among children and adolescents, taking the pattern of preparation *At home* as an outcome reference. Results adjusted for sex, area of residence, and parental education show that adolescents have lower odds of being classified in patterns characterised by high away-from-home food preparation than children: *By food retail* (OR = 0.32, 95%CI = 0.20, 0.53) and *By restaurants, canteens, and other away-from-home establishments* (OR = 0.29, 95%CI = 0.17, 0.49). Meanwhile, all non-adults living in medium urban areas have lower odds of being classified in the pattern of food preparation *By food retail* than those living in predominantly urban areas (OR = 0.42, 95%CI = 0.19, 0.91). No significant associations between patterns of food preparation and sex or parental education are observed.

**Table 3.** Association between demographic and socio-economic characteristics and patterns of food preparation, among children and adolescents (n = 1153; 3–17 years), weighted for the distribution of the Portuguese population

	Patterns of food preparation*			
	By food retail (including food consumed *in natura*)		By restaurants, canteens, and other away-from-home establishments	
	Crude OR (95%CI)	Adjusted† OR (95%CI)	Crude OR (95%CI)	Adjusted† OR (95%CI)
**Sex**				
Women	ref	ref	ref	ref
Men	1.27 (0.76, 2.11)	1.32 (0.78, 2.25)	1.26 (0.82, 1.95)	1.30 (0.83, 2.04)
**Age group**				
Children (3–9 years)	ref	ref	ref	ref
Adolescents (10–17 years)	**0.36 (0.23, 0.57)**	**0.32 (0.20, 0.53)**	**0.31 (0.19, 0.51)**	**0.29 (0.17, 0.49)**
**Area of residence**				
Predominantly urban	ref	ref	ref	ref
Medium urban	**0.47 (0.23, 0.99)**	**0.42 (0.19, 0.91)**	0.81 (0.43, 1.52)	0.69 (0.35, 1.35)
Predominantly rural	0.79 (0.39, 1.58)	0.71 (0.34, 1.48)	0.88 (0.44, 1.74)	0.93 (0.46, 1.88)
**Education** ^**‡**^				
No education/primary	ref	ref	ref	ref
Secondary	0.94 (0.41, 2.18)	0.67 (0.28, 1.61)	0.76 (0.38, 1.53)	0.69 (0.35, 1.37)
Tertiary	0.81 (0.33, 1.99)	0.61 (0.24, 1.52)	0.75 (0.38, 1.48)	0.56 (0.27, 1.16)

* Outcome reference is food preparation pattern *At home*.

† Model adjusted for sex, age group, area of residence, and education completed by parents.

‡ Education completed by parents.

#### Adults and older adults

Table 4 depicts the associations between demographic and socio-economic variables and patterns of food preparation among adults and older adults in Portugal, taking the pattern of food preparation *At home by themselves* as outcome reference, before and after adjusting for sex, age group, area of residence, and education as appropriate. Results show that, irrespectively of age group, adult men have higher odds of being classified in patterns characterised by high intakes of food prepared by others when compared to women: *At home by relatives or friends* (OR = 11.60, 95%CI = 7.58, 17.75), *By food retail* (OR = 2.05, 95%CI = 1.58, 2.66), and *By restaurants, canteens, and other away-from-home establishments* (OR = 4.20, 95%CI = 3.17, 5.57). On the other hand, compared to younger adults, older adults have lower odds of being classified in patterns of food preparation *At home by relatives or friends* (OR = 0.57, 95%CI = 0.37, 0.89) and *By restaurants, canteens, and other away-from-home establishments* (OR = 0.37, 95%CI = 0.26, 0.53), but not *By food retail* (OR = 0.90, 95%CI = 0.64, 1.26). Furthermore, all adults living in predominantly rural areas have lower odds of falling in the pattern of food preparation *By restaurants, canteens, and other away-from-home establishments* than those living in predominantly urban ones (OR = 0.58, 95%CI = 0.38, 0.88). Conversely, all adults living in households with children or adolescents have higher odds of falling in this pattern (OR = 1.52, 95%CI = 1.10, 2.11).

**Table 4.** Association between demographic and socio-economic characteristics and patterns of food preparation, among adults and older adults (n = 3852; 18–84 years), weighted for the distribution of the Portuguese population

	Patterns of food preparation*					
	At home by relatives or friends		By food retail (including food consumed *in natura*)		By restaurants, canteens, and other away-from-home establishments	
	Crude OR (95%CI)	Adjusted† OR (95%CI)	Crude OR (95%CI)	Adjusted† OR (95%CI)	Crude OR (95%CI)	Adjusted† OR (95%CI)
**Sex**						
Women	ref	ref	ref	ref	ref	ref
Men	**10.48 (6.97, 15.74)**	**11.60 (7.58, 17.75)**	**1.99 (1.53, 2.58)**	**2.05 (1.58, 2.66)**	**3.72 (2.89, 4.79)**	**4.20 (3.17, 5.57)**
**Age group**						
Adults (18–64 years)	ref	ref	ref	ref	ref	ref
Older adults (65–84 years)	**0.45 (0.30, 0.67)**	**0.57 (0.37, 0.89)**	**0.62 (0.44, 0.86)**	0.90 (0.64, 1.26)	**0.21 (0.15, 0.29)**	**0.37 (0.26, 0.53)**
**Area of residence**						
Predominantly urban	ref	ref	ref	ref	ref	ref
Medium urban	1.28 (0.92, 1.78)	1.05 (0.68, 1.63)	0.76 (0.52, 1.12)	0.83 (0.58, 1.20)	0.87 (0.67, 1.12)	0.94 (0.68, 1.31)
Predominantly rural	1.05 (0.62, 1.80)	0.93 (0.47, 1.86)	0.60 (0.35, 1.05)	0.62 (0.35, 1.12)	**0.58 (0.37, 0.90)**	**0.58 (0.38, 0.88)**
**Education**						
No education/primary	ref	ref	ref	ref	ref	ref
Secondary	**2.38 (1.66, 3.41)**	**2.32 (1.55, 3.47)**	**2.72 (1.95, 3.78)**	**2.58 (1.85, 3.59)**	**4.54 (3.23, 6.37)**	**3.29 (2.27, 4.76)**
Tertiary	**1.98 (1.35, 2.92)**	**2.31 (1.31, 4.10)**	**3.01 (1.89, 4.79)**	**2.85 (1.79, 4.53)**	**7.27 (4.82, 10.96)**	**5.48 (3.59, 8.37)**
**Marital status**						
Not married	ref	ref	ref	ref	ref	ref
Married/cohabiting	0.93 (0.62, 1.39)	0.76 (0.46, 1.28)	0.83 (0.60, 1.15)	0.90 (0.64, 1.25)	**0.73 (0.54, 0.98)**	0.80 (0.59, 1.09)
**Household composition**						
Without children/adolescents	ref	ref	ref	ref	ref	ref
With children/adolescents	**1.56 (1.00, 2.43)**	1.38 (0.76, 2.49)	**1.67 (1.21, 2.30)**	1.31 (0.92, 1.87)	**2.19 (1.63, 2.94)**	**1.52 (1.10, 2.11)**
**Household monthly net income**						
≤ €970	ref	ref	ref	ref	ref	ref
€971 – 1 940	**1.62 (1.06, 2.48)**	**1.83 (1.18, 2.82)**	**2.24 (1.62, 3.09)**	**2.15 (1.55, 2.99)**	**2.68 (2.01, 3.57)**	**2.45 (1.81, 3.31)**
> €1 940	**2.40 (1.42, 4.05)**	**2.13 (1.25, 3.60)**	**4.03 (2.44, 6.63)**	**3.60 (2.14, 6.05)**	**6.39 (4.19, 9.74)**	**4.59 (2.99, 7.04)**
**Household food insecurity status**						
Food security	ref	ref	ref	ref	ref	ref
Food insecurity (mild and severe)	**0.53 (0.34, 0.83)**	**0.43 (0.27, 0.69)**	**0.53 (0.36, 0.78)**	**0.63 (0.42, 0.95)**	**0.36 (0.26, 0.52)**	**0.53 (0.34, 0.82)**

* Outcome reference is food preparation pattern *At home by themselves*.

† Models adjusted for sex, age group, area of residence, and education, except for household monthly net income, where it was adjusted for sex, age group, and area of residence only.

Findings in Table 4 also reveal that, irrespectively of age group, adults with secondary or tertiary education have higher odds of being classified in patterns characterised by high intakes of food prepared by others than those completing none or primary education only: *At home by relatives or friends* (secondary: OR = 2.32, 95%CI = 1.55, 3.47; tertiary: OR = 2.31, 95%CI = 1.31, 4.10), *By food retail* (secondary: OR = 2.58, 95%CI = 1.85, 3.59; tertiary: OR = 2.85, 95%CI = 1.79, 4.53), and *By restaurants, canteens, and other away-from-home establishments* (secondary: OR = 3.29, 95%CI = 2.27, 4.76; tertiary: OR = 5.48, 95%CI = 3.59, 8.37). Likewise, adults from households with a monthly net income higher than €970 have higher odds of falling in these patterns than those in a lower income range: *At home by relatives or friends* (€971–1940: OR=1.83, 95%CI = 1.18, 2.82; >€1940: OR = 2.13, 95%CI = 1.25, 3.60), *By food retail* (€971–1940: OR = 2.15, 95%CI = 1.55, 2.99; > €1940: OR = 3.60, 95%CI = 2.14, 6.05), and *By restaurants, canteens, and other away-from-home establishments* (€971–1940: OR = 2.45, 95%CI = 1.81, 3.31; >€1940: OR = 4.59, 95%CI = 2.99, 7.04). On the other hand, all adults from food-insecure households have lower odds of being classified in these patterns than the remainder: *At home by relatives or friends* (OR = 0.43, 95%CI = 0.27, 0.69), *By food retail* (OR = 0.63, 95%CI = 0.42, 0.95), and *By restaurants, canteens, and other away-from-home establishments* (OR = 0.53, 95%CI = 0.34, 0.82). No significant associations between patterns of food preparation and marital status are found after adjustment for other variables.

### Health and nutritional status, physical activity, and diet quality

#### Children and adolescents

Table 5 shows the associations between patterns of food preparation among children and adolescents and their health and nutritional status, as well as diet quality. No significant associations are observed between the patterns and the prevalence of NCDs or overweight/obesity. Notwithstanding, children and adolescents falling in the pattern of preparation *By food retail* exhibit higher odds of having a low diet quality (OR = 2.58, 95%CI = 1.46, 4.54) than those falling in the pattern of preparation *At home*.

**Table 5.** Association between patterns of food preparation and health and nutritional status and diet quality, among children and adolescents (n = 1153; 3–17 years), weighted for the distribution of the Portuguese population

	Health status (NCDs) No (ref) vs. yes	
	Model 1* OR (95%CI)	Model 2† OR (95%CI)
**Patterns of food preparation**		
*At home*	ref	ref
*By food retail* (including food consumed *in natura*)	0.77 (0.42, 1.44)	0.83 (0.46, 1.50)
*By restaurants, canteens, and other away-from-home establishments*	0.79 (0.41, 1.53)	0.85 (0.45, 1.59)

	Nutritional status (BMI) Normal/underweight (ref) vs. overweight and obesity	
	Model 1* *OR (95%CI)*	Model 2† *OR (95%CI)*
**Patterns of food preparation**		
*At home*	ref	ref
*By food retail* (including food consumed *in natura*)	1.43 (0.76, 2.66)	1.37 (0.72, 2.59)
*By restaurants, canteens, and other away-from-home establishments*	1.17 (0.71, 1.90)	1.15 (0.70, 1.87)

	Diet quality (MAI) Medium and high (ref) vs. low	
	Model 1* *OR (95%CI)*	Model 2† *OR (95%CI)*
**Patterns of food preparation**		
*At home*	ref	ref
*By food retail* (including food consumed *in natura*)	**2.78 (1.59, 4.87)**	**2.58 (1.46, 4.54)**
*By restaurants, canteens, and other away-from-home establishments*	**1.71 (1.04, 2.82)**	1.65 (0.98, 2.77)

NCDs, noncommunicable diseases; MAI, Mediterranean Adequacy Index.

* Adjusted for total energy intake.

† Adjusted for total energy intake, sex, age group, area of residence, and education completed by parents.

#### Adults and older adults

Table 6 shows the associations between the patterns of food preparation among adults and older adults and their health and nutritional status, level of physical activity, and diet quality. No associations between patterns of food preparation and the prevalence of NCDs remain after controlling for sex, age group, area of residence, and education. Yet, adults and older adults falling in the pattern of preparation *By restaurants, canteens, and other away-from-home establishments* present lower odds of overweight/obesity (OR = 0.74, 95%CI = 0.56, 0.97) than those falling in the pattern of preparation *At home by themselves*.

**Table 6.** Association between patterns of food preparation and health and nutritional status, level of physical activity, and diet quality, among adults and older adults (n = 3852; 18–84 years), weighted for the distribution of the Portuguese population

	Health status (NCDs) No (ref) vs. yes	
	Model 1* OR (95%CI)	Model 2† OR (95%CI)
**Patterns of food preparation**		
*At home by themselves*	ref	ref
*At home by relatives or friends*	0.78 (0.54, 1.11)	1.00 (0.68, 1.47)
*By food retail* (including food consumed *in natura*)	0.80 (0.60, 1.07)	1.08 (0.76, 1.52)
*By restaurants, canteens, and other away-from-home establishments*	**0.54 (0.41, 0.72)**	0.91 (0.69, 1.20)

	Nutritional status (BMI) Normal/underweight (ref) vs. overweight and obesity	
	Model 1* *OR (95%CI)*	Model 2† *OR (95%CI)*
**Patterns of food preparation**		
*At home by themselves*	ref	ref
*At home by relatives or friends*	0.72 (0.49, 1.05)	0.71 (0.47, 1.08)
*By food retail* (including food consumed *in natura*)	**0.63 (0.46, 0.87)**	0.78 (0.54, 1.11)
*By restaurants, canteens, and other away-from-home establishments*	**0.55 (0.44, 0.70)**	**0.74 (0.56, 0.97)**

	Physical activity (IPAQ) Active and minimally active (ref) vs. inactive	
	Model 1* *OR (95%CI)*	Model 2† *OR (95%CI)*
**Patterns of food preparation**		
*At home by themselves*	ref	ref
*At home by relatives or friends*	**1.64 (1.18, 2.29)**	**1.84 (1.32, 2.57)**
*By food retail* (including food consumed *in natura*)	1.09 (0.78, 1.52)	1.14 (0.81, 1.60)
*By restaurants, canteens, and other away-from-home establishments*	1.31 (0.99, 1.72)	**1.45 (1.08, 1.96)**

	Diet quality (MAI) Medium and high (ref) vs. low	
	Model 1* *OR (95%CI)*	Model 2† *OR (95%CI)*
**Patterns of food preparation**		
*At home by themselves*	ref	ref
*At home by relatives or friends*	**2.06 (1.27, 3.33)**	**2.03 (1.25, 3.31)**
*By food retail* (including food consumed *in natura*)	**3.91 (2.59, 5.90)**	**3.59 (2.36, 5.45)**
*By restaurants, canteens, and other away from home establishments*	**3.54 (2.46, 5.10)**	**3.01 (2.08, 4.34)**

NCDs, noncommunicable diseases; IPAQ, International Physical Activity Questionnaire; MAI, Mediterranean Adequacy Index.

* Adjusted for total energy intake.

† Adjusted for total energy intake, sex, age group, area of residence, and education.

Findings in Table 6 also uncover that adults and older adults falling in the patterns of preparation *At home by relatives or friends* (OR = 1.84, 95%CI = 1.32, 2.57) or *By restaurants, canteens, and other away-from-home establishments* (OR = 1.45, 95%CI = 1.08, 1.96) have higher odds of reporting low physical activity than those falling in the pattern of preparation *At home by themselves*. Moreover, adults and older adults falling in patterns characterised by high intakes of foods prepared by others show higher odds of having low diet quality (*At home by relatives or friends*: OR = 2.03, 95%CI = 1.25, 3.31; *By food retail*: OR = 3.59, 95%CI = 2.36, 5.45; *By restaurants, canteens, and other away-from-home establishments*: OR = 3.01, 95%CI = 2.08, 4.34).

## Discussion

Studies investigating the prevalence of at-home versus away-from-home patterns of food preparation at population level remain scarce, particularly those drawing from the results of dietary intake assessments of nationally representative samples encompassing individuals of all ages.^(24,36–38)^ This prevents the development of a better understanding of the factors driving the consumption of food prepared at home versus away from home, as well as of related effects on nutrition and health. The present work contributes to close this gap by performing a cross-sectional analysis of food intake data drawn from IAN-AF 2015/16 to identify patterns of food preparation for both children and adults in Portugal and uncover their associations with demographic and socio-economic characteristics, health and nutritional status, level of physical activity, and diet quality. Our main results indicate that the most common pattern of food preparation involves a high intake of foods and beverages prepared by foodservice establishments as restaurants and canteens. They also indicate that higher socio-economic status is linked to greater reliance on foods prepared away from home, which impacts on a poorer diet quality and a more sedentary lifestyle, but not necessarily on higher obesity rates or NCDs. These findings support the need for tailored public health strategies that consider the most vulnerable groups, and provide valuable insights for developing more effective public policies aimed at increasing access to healthy food options in foodservice establishments as well as promoting more active lifestyles among people less likely to cook at home.

### Patterns of food preparation

Regarding food preparation patterns, the findings of this study indicate that nearly three-quarters (71.7%) of Portuguese residents eat foods and beverages prepared and/or procured mainly away from home, that is, originating from the foodservice or the food processing and retail sectors rather than prepared at home. Indeed, household expenditure data place the share of away-from-home food spending in Portugal at 10.6% in 2022, well above the European Union average of 6.7%.^(56)^ Despite Portugal’s disposable income per capita being lower than the European Union average, work schedules, better weather conditions, and cultural preferences for dining out, together with the increasing availability and convenience of foodservice options may contribute to the Portuguese higher spending on food away from home. A comparable analysis of adult food intake data from the United Kingdom National Diet and Nutrition Survey 2008/16 reveals the consumption of food prepared at home to be low.^(24)^ On the contrary, analyses of adult intakes from the North/South Ireland Food Consumption Survey 1997/99,^(37)^ the US National Health and Nutrition Examination Surveys 2003/04 and 2007/08,^(6)^ the Republic of Korea National Health and Nutrition Examination Survey 2007/09,^(29)^ as well as of intakes from the Republic of Ireland National Children’s Food Survey 2003/04,^(36)^ indicate that the consumption of food prepared at home is largely prevalent in these regions. Aside from variation in food preferences and habits attributable to culture, such disparity in extant findings likely stems from underlying methodological differences in the timeframe, sampling, age group, dietary assessment protocol, and food intake classification applied.^(57,58)^

### Demographic and socio-economic characteristics

Respecting individual demographic characteristics, the results of this study show that adult men are less likely than women to consume foods prepared at home by themselves, underscoring their traditional lower involvement in domestic cooking and suggesting a possibly higher reliance on others for food preparation and supply.^(59)^ Adult men are also found to consume less home-prepared food than women in the United Kingdom, albeit the difference here being small.^(24)^ Similarly, adult men have lower odds to eat meals prepared at home than women in the Republic of Korea.^(29,30)^ Meanwhile, in the present study, adolescents are more likely than children to consume foods prepared at home by themselves, relatives, or friends. Being more autonomous and independent than younger children, adolescents may more often leave school and return home at noon to eat a cooked lunch, rather than doing so in school premises.^(60,61)^ This might explain why they eat relatively more food prepared at home. In the Republic of Ireland, where a cooked lunch is generally not provided at primary schools, older children (9–12 years) are found to be more likely to eat food prepared away from home than younger children (5–8 years), particularly products acquired from food retail and takeaway outlets.^(36)^ Furthermore, older adults in the present study are less likely to consume foods prepared at home by relatives or friends or prepared at restaurants, canteens, and other away-from-home establishments, than other adults, indicating a likely higher involvement in grocery shopping and domestic food preparation activities. Lower time and effort demands from work-related activities and the loss of income that accompany ageing and retirement probably explain why older adults resort more often to food prepared at home.^(62)^ For instance, younger adults tend to consume more commercially prepared meals than older adults in the Republic of Korea.^(30)^ Nevertheless, the consumption of home-prepared food by older adults in the United Kingdom is found to be similar to that of younger adults.^(24)^ Lastly, although married/cohabiting adults are found to have higher odds of eating meals prepared at home than unmarried ones in the Republic of Korea,^(29,30)^ no associations between marital status and patterns of food preparation are observed in the present study.

Regarding household demographics, findings show that adults and older adults living in predominantly rural areas, as well as children and adolescents living in medium urban areas, are less likely to consume foods prepared away from home (by foodservice and food retail, respectively) than those living in predominantly urban areas. In line with this, European adults with the highest access to restaurants and medium access to grocery stores, both typical of predominantly urban areas, are shown to have the lowest likelihood of preparing meals at home daily.^(63)^ Moreover, the adults and older adults in the present study who live in households with children or adolescents are more likely to consume food prepared by restaurants, canteens, and other away-from-home establishments than the remainder. Similarly, results from the US Department of Agriculture’s 2012/13 National Household Food Acquisition and Purchase Survey show that the presence of children (<18 years) in households increases their expenditures in fast-food and full-service restaurants.^(64)^ The food provisioning strategies of contemporary families may be a relevant explanation for the higher consumption of meals from out-of-home sources by households with children or adolescents. In particular, the purchase of convenience foods and ready-to-eat meals may be favoured as the consumption of such products typically lowers preparation, cooking, and kitchen clean-up times.^(65)^

With respect to socio-economic variables, the adults and older adults with higher education or from households with higher income in this study are more likely to consume food prepared away from home than the remainder, whereas the opposite is true for those living in food-insecure households. High-income adults in the United States,^(6)^ as well as adults who are employed, more educated, and of a higher economic status in the Republic of Korea,^(29,30)^ equally tend to consume less food from home sources. Conversely, university-graduated adults in the United Kingdom tend to eat slightly more home-prepared food.^(24)^ All evidence considered, the balance between at-home and away-from-home food consumption is likely to constitute a fairly good indicator of the type and rate of socio-economic development of regions and countries; the distribution of income, labour, time, and expense within and between households; and the prevalence of gender and social inequalities in societies.^(26,66)^

### Health and nutritional status, physical activity, and diet quality

Concerning nutritional and health status, the results of this study indicate that adults and older adults with higher consumption of food prepared by restaurants and other foodservice establishments are less likely to be overweight or obese than those more reliant on the food they prepare at home themselves. There are further no associations between food preparation patterns and weight or health status after adjustment for total energy intake, sex, age group, area of residence, and education. Extant systematic reviews and meta-analyses generally conclude for the existence of a positive link between the consumption of food away-from-home consumption and markers of NCDs, particularly BMI.^(15,57,67)^ However, they also highlight that high variation as well as important limitations in how associations between eating out, diet quality, and nutritional status are typically studied weaken such conclusion,^(68)^ with several cross-sectional and prospective cohort studies finding no or negative associations.^(57,58,67)^ On the other hand, cohort studies conducted in the United States and the United Kingdom suggest there is a preventive effect of increasing the frequency of consumption of homecooked meals, at the expense of eating out, on the risk of overweight, obesity, and type 2 diabetes, likely to be mediated by an improvement of diet quality.^(34,69,70)^ Findings from the present study, as well as from a French prospective cohort,^(31)^ do not support these hypotheses, however. Furthermore, several studies point out that the protective effects of increasing engagement in meal preparation activities at home on diet and health may be higher for household members than caregivers themselves.^(71–74)^ Meanwhile, aspects related to disposable income and lifecycle, such as household income class, size and composition, and engagement in the workforce are likely important moderators of the effects of food preparation and consumption practices on diet quality and BMI.^(75–77)^ Beyond the regression analyses conducted in the present study, future studies should address the roles played by the aforementioned mediating and moderating factors.

With respect to lifestyle factors, the present study shows that, irrespectively of age group, individuals with a high intake of food they do not prepare themselves (i.e. meals prepared at home by relatives or friends or away-from-home by restaurants, canteens, and other foodservice establishments) are less likely to adhere to the Mediterranean diet and more likely to report low physical activity. Somewhat in line with this, adherence to DASH (Dietary Approaches to Stopping Hypertension) in the United Kingdom is linked to higher consumption of homecooked food among adults.^(24)^ Noticeably, a previous pan-European study has also found evidence of a positive association between eating out of home and sedentarism.^(42)^ An important gap in research relating food preparation and consumption practices with physical activity remains, nonetheless, as most studies so far look exclusively at associations with diet quality and nutritional status.^(17)^

### Strengths and limitations

To the best of the authors’ knowledge, this is the latest of the very few studies to date that draw on a cross-sectional analysis of nationally representative food intake data, from both adults and non-adults, to investigate the demographic and socio-economic determinants of food preparation practices, as well as the putative associations of such practices with nutritional status, the prevalence of NCDs, and lifestyle choices.

Further longitudinal research is necessary to determine the direction of any causation between patterns of food preparation and correlates uncovered. Improving diet quality and increasing physical activity are essentially volitional, short-term behaviours, while the effects of such lifestyle changes on weight and, subsequently, health status are much less under the control of individuals and span across the course of years or even decades. This might help explain why, in the present study, a higher consumption of food prepared out of home, or at home by caregivers, correlates to poor diet quality and sedentarism, but not necessarily to a heightened risk of overweight/obesity or chronic NCDs. Historic individual data on nutritional status, any prior attempts to modify it (e.g. by dieting, increasing exercise and other means), and related outcomes are also likely to impact present-day food preparation and consumption choices and should thus be duly accounted for in future studies.^(68)^

The use of data from the IAN–AF 2015/16, which are now 8–9 years old, may not necessarily reflect the current situation in Portugal, especially after the onset of the COVID-19 pandemic. The pandemic may have significantly altered the dietary habits of the Portuguese population, potentially increasing at-home consumption due to lockdown and social distancing measures. Nevertheless, these are the most recent and comprehensive data from Portugal we are aware of on this topic. The MAI has not been validated in Portugal; however, this index has been indicated as good as the most utilised indexes or scores in Europe.^(44)^ Even though residual confounding remains possible, logistic regression models were adjusted for a number of relevant potential confounders. Moreover, this survey followed a harmonised methodology of dietary assessment proposed by the EFSA,^(78)^ allowing the collection of extremely detailed food consumption-related variables — which, consequently, enabled us to explore the food and beverage preparation places, regardless of the absence of a descriptor variable that exclusively had evaluated it. The food and beverage preparation place classification applied in this study has the advantage of internal consistency, because consistently defines what is prepared at home or away from home for all participants, based on data collected about food consumption — which may be less subject to desirability bias than the data collected about cooking behaviour (such as one’s ability to complete specific a priori food preparations or cooking frequency), mostly used in other studies.^(26–28,66,79)^ In addition, our system classification was informed by previously literature,^(20,24)^ and this approach, in contrast to considering only the place of consumption reported for each eating occasion, holds the potential to acknowledge the intake of foods and beverages at home that were not homemade.

## Conclusions

We undertook a broad and largely generalisable exploration of the demographic and socio-economic determinants of food preparation practices in a European country where such analysis was lacking. Our findings contribute to the state of the art on the antecedents of food preparation practices and their likely impacts on diet, nutrition, and health. Specifically, they suggest that the eating patterns of certain population groups — adult men, with higher education, from multigenerational households with higher income — are prone to be composed mainly of foods and beverages prepared out of home or at home by caregivers and often linked to poor diet quality and sedentarism. Meanwhile, self-prepared food dominates the meals of more vulnerable population groups — adolescents, older adults, adults residing in rural areas, and those belonging to food-insecure households — being related to higher adherence to Mediterranean diet standards and a more active lifestyle.

However, our findings also reinforce that the pathways between food preparation practices, diet, weight, and health at the population level are highly complex and possibly less intuitive than might be expected. This calls for continued research in diverse geographies, grounded on socioecological approaches to the prevention of overweight/obesity and NCDs, as well as on more robust methodology and evidence. In particular, giving a more central role to the promotion of home cooking in future public health nutrition interventions and dietary recommendations should be undertaken with caution, as it may represent yet another burden laid on already vulnerable groups and may not translate into the expected long term health benefits to the general population.

## Supporting information

Rei et al. supplementary material Rei et al. supplementary material

## Supplementary material

The supplementary material for this article can be found at https://doi.org/10.1017/jns.2024.87.

## References

[1] Roth GA , Abate D , Abate KH et al. Global, regional, and national age-sex-specific mortality for 282 causes of death in 195 countries and territories, 1980–2017: a systematic analysis for the global burden of disease study 2017. The Lancet. 2018;392;(10159):1736–1788. doi: 10.1016/S0140-6736(18)32203-7 PMC6227606 30496103

[2] World Health Organization Noncommunicable Diseases Country Profiles 2018. Geneva: WHO; 2018.

[3] World Health Organization Noncommunicable Diseases: Progress Monitor 2022. Geneva: WHO; 2022.

[4] Fanzo J , Davis C Can diets be healthy, sustainable, and equitable? Curr Obes Rep. 2019;8(4):495–503. doi: 10.1007/s13679-019-00362-0 31654336 PMC6910888

[5] Möser A () Food preparation patterns in German family households. An econometric approach with time budget data. Appetite. 2010;55(1):99–107. doi: 10.1016/j.appet.2010.04.008 20420870

[6] Smith LP , Ng SW , Popkin BM Trends in US home food preparation and consumption: analysis of national nutrition surveys and time use studies from 1965-1966 to 2007-2008. Nutr J. 2013;12:45. doi: 10.1186/1475-2891-12-45 23577692 PMC3639863

[7] Jabs J & Devine CM Time scarcity and food choices: an overview. Appetite. 2006;47(2):196–204. doi: 10.1016/j.appet.2006.02.014 16698116

[8] Venn D & Strazdins L Your money or your time? How both types of scarcity matter to physical activity and healthy eating. Soc Sci Med. 2017;172:98–106. doi: 10.1016/j.socscimed.2016.10.023 27839899

[9] Jabs J , Devine CM , Bisogni CA et al.) Trying to find the quickest way: employed mothers’ constructions of time for food. J Nutr Educ Behav. 2007;39(1):18–25. doi: 10.1016/j.jneb.2006.08.011 17276323

[10] Jekanowski MD Causes and consequences of fast food sales growth. Food Rev/ Nat Food Rev. 1999;22:1. doi: 10.22004/ag.econ.266201

[11] Hawkes C Marketing activities of global soft drink and fast food companies in emerging markets: a review. In Globalization, Diets and Noncommunicable Diseases [World Health Organization, editor]. 2002.

[12] Cheng SL , Olsen W , Southerton D et al. The changing practice of eating: evidence from UK time diaries, 1975 and 2000. Br J Sociol. 2007;58(1):39–61. doi: 10.1111/j.1468-4446.2007.00138.x 17343637

[13] Lang T , Caraher M Is there a culinary skills transition? Data and debate from the UK about changes in cooking culture. J Home Econ Inst Aust. 2001;8(2):2–14.

[14] Lachat C , Nago E , Verstraeten R et al. Eating out of home and its association with dietary intake: a systematic review of the evidence. Obes Rev. 2012;13(4):329–346. doi: 10.1111/j.1467-789X.2011.00953.x 22106948

[15] Nago ES , Lachat CK , Dossa RA et al. Association of out-of-home eating with anthropometric changes: a systematic review of prospective studies. Crit Rev Food Sci Nutr. 2014;54(9):1103–1116. doi: 10.1080/10408398.2011.627095 24499144

[16] Lam MCL & Adams J Association between home food preparation skills and behaviour, and consumption of ultra-processed foods: Cross-sectional analysis of the UK National Diet and nutrition survey (2008–2009). Int J Behav Nutr Phys Act. 2017;14(1):68. doi: 10.1186/s12966-017-0524-9 28535769 PMC5442685

[17] Mills S , White M , Brown H et al. Health and social determinants and outcomes of home cooking: A systematic review of observational studies. Appetite. 2017;111:116–134. doi: 10.1016/j.appet.2016.12.022 28024883

[18] Clifford Astbury C , Penney TL , Adams J. Comparison of individuals with low versus high consumption of home-prepared food in a group with universally high dietary quality: a cross-sectional analysis of the UK National Diet & Nutrition Survey (2008–2016). Int J Behav Nutr Phys Act. 2019;16(1):9. doi: 10.1186/s12966-019-0768-7 30654805 PMC6337812

[19] Farfán G , Genoni ME , Vakis R You are what (and where) you eat: Capturing food away from home in welfare measures. Food Policy. 2017;72:146–156. doi: 10.1016/j.foodpol.2017.08.020

[20] Costa AIA , Dekker M , Beumer RR et al. A consumer-oriented classification system for home meal replacements. Food Qual Prefer. 2001;12(4):229–242. doi: 10.1016/S0950-3293(01)00010-6

[21] Peltner J & Thiele S Convenience-based food purchase patterns: identification and associations with dietary quality, sociodemographic factors and attitudes. Public Health Nutr. 2018;21(3):558–570. doi: 10.1017/s1368980017003378 29173221 PMC10261000

[22] Adams J , Goffe L , Brown T et al. Frequency and socio-demographic correlates of eating meals out and take-away meals at home: cross-sectional analysis of the UK national diet and nutrition survey, waves 1–4 (2008–12). Int J Behav Nutr Phys Act. 2015;12(1):51. doi: 10.1186/s12966-015-0210-8 25889159 PMC4404110

[23] Goffe L , Rushton S , White M et al. Relationship between mean daily energy intake and frequency of consumption of out-of-home meals in the UK national diet and nutrition survey. Int J Behav Nutr Phys Act. 2017;14(1):131. doi: 10.1186/s12966-017-0589-5 28938893 PMC5610411

[24] Clifford Astbury C , Penney TL , Adams J Home-prepared food, dietary quality and socio-demographic factors: a cross-sectional analysis of the UK national diet and nutrition survey 2008–16. Int J Behav Nutr Phys Act. 2019;16(1):82. doi: 10.1186/s12966-019-0846-x 31492141 PMC6729029

[25] Powell LM , Nguyen BT , Han E Energy intake from restaurants: demographics and socioeconomics, 2003-2008. Am J Prev Med. 2012;43(5):498–504. doi: 10.1016/j.amepre.2012.07.041 23079172 PMC3479669

[26] Virudachalam S , Long JA , Harhay MO et al. Prevalence and patterns of cooking dinner at home in the USA: National Health and Nutrition Examination Survey (NHANES) 2007-2008. Public Health Nutr. 2014;17(5):1022–1030. doi: 10.1017/s1368980013002589 24107577 PMC10282260

[27] Wolfson JA & Bleich SN () Is cooking at home associated with better diet quality or weight-loss intention? Public Health Nutr. 2015;18(8):1397–1406. doi: 10.1017/s1368980014001943 25399031 PMC8728746

[28] Wolfson JA , Leung CW , Richardson CR More frequent cooking at home is associated with higher Healthy Eating Index-2015 score. Public Health Nutr. 2020;23(13):2384–2394. doi: 10.1017/S1368980019003549 31918785 PMC11374573

[29] Lee KW , Song WO , Cho MS Dietary quality differs by consumption of meals prepared at home vs. outside in Korean adults. Nutr Res Pract. 2016;10(3):294–304. doi: 10.4162/nrp.2016.10.3.294 27247726 PMC4880729

[30] Choi I , Kim WG , Yoon J Energy intake from commercially-prepared meals by food source in Korean adults: Analysis of the 2001 and 2011 Korea national health and nutrition examination surveys. Nutr Res Pract. 2017;11(2):155–162. doi: 10.4162/nrp.2017.11.2.155 28386389 PMC5376534

[31] Méjean C , Lampuré A , Si Hassen W et al. Influence of food preparation behaviors on 5-year weight change and obesity risk in a French prospective cohort. Int J Behav Nutr Phys Act. 2018;15(1):120. doi: 10.1186/s12966-018-0747-4 30477513 PMC6258165

[32] Méjean C , Si Hassen W , Gojard S et al. Social disparities in food preparation behaviours: a DEDIPAC study. Nutr J. 2017;16(1):62. doi: 10.1186/s12937-017-0281-2 28931416 PMC5607511

[33] Mills S , Adams J , Wrieden W et al. () Sociodemographic characteristics and frequency of consuming home-cooked meals and meals from out-of-home sources: cross-sectional analysis of a population-based cohort study. Public Health Nutr. 2018;21(12):2255–2266. doi: 10.1017/S1368980018000812 29637874 PMC6064641

[34] Mills S , Brown H , Wrieden W et al. Frequency of eating home cooked meals and potential benefits for diet and health: cross-sectional analysis of a population-based cohort study. Int J Behav Nutr Phys Act. 2017;14(1):109. doi: 10.1186/s12966-017-0567-y 28818089 PMC5561571

[35] Díaz-Méndez C & García-Espejo I Eating practice models in Spain and the United Kingdom: a comparative time-use analysis. Int J Comp Sociol. 2014;55(1):24–44. doi: 10.1177/0020715213519657

[36] Burke SJ , McCarthy SN , O’Neill JL et al. An examination of the influence of eating location on the diets of Irish children. Public Health Nutr. 2007;10(6):599–607. doi: 10.1017/s1368980007258379 17381926

[37] O’Dwyer NA , Gibney MJ , Burke SJ et al. The influence of eating location on nutrient intakes in Irish adults: implications for developing food-based dietary guidelines. Public Health Nutr. 2005;8(3):258–265. doi: 10.1079/phn2004701 15918922

[38] O’Dwyer NA , McCarthy SN , Burke SJ et al. The temporal pattern of the contribution of fat to energy and of food groups to fat at various eating locations: implications for developing food-based dietary guidelines. Public Health Nutr. 2005;8(3):249–257. doi: 10.1079/phn2004700 15918921

[39] Perista H , Cardoso A , Brázia A et al. *The Use of Time by Men and Women in Portugal*. CESIS – Centro de Estudos para a Intervenção Social, CITE – Comissão para a Igualdade no Trabalho e no Emprego. 2016.

[40] Naska A , Katsoulis M , Orfanos P et al. Eating out is different from eating at home among individuals who occasionally eat out. A cross-sectional study among middle-aged adults from eleven European countries. Br J Nutr. 2015;113(12):1951–1964. doi: 10.1017/S0007114515000963 25907775

[41] Orfanos P , Naska A , Rodrigues S et al. Eating at restaurants, at work or at home. Is there a difference? A study among adults of 11 European countries in the context of the HECTOR* project. Eur J Clin Nutr. 2017;71(3):407–419. doi: 10.1038/ejcn.2016.219 27966568

[42] Orfanos P , Naska A , Trichopoulos D et al. Eating out of home and its correlates in 10 European countries. The European prospective investigation into cancer and nutrition (EPIC) study. Public Health Nutr. 2007;10(12):1515–1525. doi: 10.1017/S1368980007000171 17582244

[43] Lopes C , Torres D , Oliveira A et al. () National food, nutrition, and physical activity survey of the Portuguese general population (2015-2016): protocol for design and development. JMIR Res Protoc. 2018;7(2):e42. doi: 10.2196/resprot.8990 29449204 PMC5832902

[44] Alberti A , Fruttini D , Fidanza F The Mediterranean adequacy index: further confirming results of validity. Nutr Metab Cardiovasc Dis. 2009;19(1):61–66. doi: 10.1016/j.numecd.2007.11.008 18337072

[45] Instituto Nacional de Estatística 2014 Statistical divisions - Classification of urban areas https://www.ine.pt/xportal/xmain?xpid=INE&xpgid=ine_cont_inst&INST=6251013&xlang=en (accessed June 2022).

[46] Radimer KL , Olson CM , Campbell CC () Development of indicators to assess hunger. J Nutr. 1990;120(Suppl 11): 1544–1548. doi: 10.1093/jn/120.suppl_11.1544 2243303

[47] World Health Organization Expert Committee Physical Status: The Use of and Interpretation of Anthropometry. Geneva: WHO; 1995. 8594834

[48] Craig CL , Marshall AL , Sjöström M et al. () International physical activity questionnaire: 12-country reliability and validity. Med Sci Sports Exerc. 2003;35(8):1381–1395. doi: 10.1249/01.Mss.0000078924.61453.Fb 12900694

[49] Guidelines for the Data Processing and Analysis of the International Physical Activity Questionnaire (IPAQ). Short and Long Forms, November 2005. https://sites.google.com/view/ipaq/score (accessed June 2022).

[50] Goios AC , Severo M , Lloyd AJ et al. Validation of a new software eAT24 used to assess dietary intake in the adult Portuguese population. Public Health Nutr. 2020;23(17):3093–3103. doi: 10.1017/s1368980020001044 32611472 PMC10200576

[51] European Food Safety Authority The food classification and description system FoodEx 2 (draft-revision 1). EFSA Support Publ. 2011;8(12):215E. doi: 10.2903/sp.efsa.2011.EN-215 PMC13093102 42015988

[52] European Food Safety Authority The food classification and description system FoodEx 2 (revision 2). EFSA Support Publ. 2015;12(5):804E. doi: 10.2903/sp.efsa.2015.EN-804

[53] Ward JH Hierarchical grouping to optimize an objective function. J Am Stat Assoc. 1963;58(301):236–244. doi: 10.1080/01621459.1963.10500845

[54] R: A language and environment for statistical computing *. R Foundation for Statistical Computing*. 2020. http://www.r-project.org/index.html (accessed June 2022).

[55] Lumley T Analysis of complex survey samples. J Stat Softw. 2004;9(8):1–19. doi: 10.18637/jss.v009.i08

[56] Eurostat 2022 Final consumption expenditure of households by consumption purpose https://ec.europa.eu/eurostat/databrowser/view/NAMA_10_CO3_P3__custom_107542/bookmark/table?lang=en&bookmarkId=0a42eb21-a6f0-4f23-9556-58656ac77be3 (accessed June 2024).

[57] Bezerra IN , Curioni C , Sichieri R Association between eating out of home and body weight. Nutr Rev. 2012;70(2):65–79. doi: 10.1111/j.1753-4887.2011.00459.x 22300594

[58] Wellard-Cole L , Davies A , Allman-Farinelli M Contribution of foods prepared away from home to intakes of energy and nutrients of public health concern in adults: a systematic review. Crit Rev Food Sci Nutr. 2022;62(20):5511–5522. doi: 10.1080/10408398.2021.1887075 33596740

[59] Cerrato J , Cifre E Gender inequality in household chores and work-family conflict. Original Research. Front Psychol. 2018;9:1330. doi: 10.3389/fpsyg.2018.01330 30123153 PMC6086200

[60] Cordovil R , Lopes F , Neto C Children’s (in)dependent mobility in Portugal. J Sci Med Sport. 2015;18(3):299–303. doi: 10.1016/j.jsams.2014.04.013 24933503

[61] Marzi I & Reimers AK Children’s independent mobility: current knowledge, future directions, and public health implications. Int J Environ Res Public Health. 2018;15:11. doi: 10.3390/ijerph15112441 PMC6267483 30388880

[62] Crompton R & Lyonette C Work-life ‘balance’ in Europe. Acta Sociologica. 2006;49(4):379–393.

[63] Pinho MGM , Mackenbach JD , Charreire H et al. Spatial access to restaurants and grocery stores in relation to frequency of home cooking. Int J Behav Nutr Phys Act. 2018;15(1):6. doi: 10.1186/s12966-017-0640-6 29338756 PMC5771126

[64] Saksena M , Okrent A , Anekwe TD et al. America’s Eating Habits: Food Away From Home. Washington, D.C: United States Department of Agriculture, Economic Research Service; 2018.

[65] Carrigan M , Szmigin I , Leek S Managing routine food choices in UK families: the role of convenience consumption. Appetite. 2006;47(3): 372–383. doi: 10.1016/j.appet.2006.05.018 16846664

[66] Adams J , Goffe L , Adamson AJ et al. Prevalence and socio-demographic correlates of cooking skills in UK adults: cross-sectional analysis of data from the UK national diet and nutrition survey. Int J Behav Nutr Phys Act. 2015;12(1):99. doi: 10.1186/s12966-015-0261-x 26242297 PMC4524366

[67] Godbharle S , Jeyakumar A , Giri BR et al. Pooled prevalence of food away from home (FAFH) and associated non-communicable disease (NCD) markers: a systematic review and meta-analysis. J Health Popul Nutr. 2022;41(1):55. doi: 10.1186/s41043-022-00335-5 36451189 PMC9709732

[68] Mancino L , Todd J , Lin B-H Separating what we eat from where: measuring the effect of food away from home on diet quality. Food Policy. 2009;34(6):557–562. doi: 10.1016/j.foodpol.2009.09.003

[69] Taillie LS & Poti JM Associations of cooking with dietary intake and obesity among supplemental nutrition assistance program participants. Am J Prev Med. 2017;52(2s2):S151–S160. doi: 10.1016/j.amepre.2016.08.021 28109417 PMC5454383

[70] Zong G , Eisenberg DM , Hu FB et al. Consumption of meals prepared at home and risk of Type 2 diabetes: an analysis of two prospective cohort studies. PLoS Med. 2016;13:7, :e1002052. doi: 10.1371/journal.pmed.1002052 PMC4933392 27379673

[71] De Ridder K , Lebacq T , Ost C et al. Enquête de Consommation Alimentaire 2014-2015. Brussels: Institut Scientifique de Santé Publique 2016.

[72] Appelhans BM , Segawa E , Janssen I et al. Meal preparation and cleanup time and cardiometabolic risk over 14 years in the Study of Women’s Health Across the Nation (SWAN). Prev Med. 2015;71:1–6. doi: 10.1016/j.ypmed.2014.11.025 25490602 PMC4329067

[73] Kramer RF , Coutinho AJ , Vaeth E et al. Healthier home food preparation methods and youth and caregiver psychosocial factors are associated with lower BMI in African American youth. J Nutr. 2012;142(5):948–954. doi: 10.3945/jn.111.156380 22457390

[74] Chu YL , Addo OY , Perry CD et al. Time spent in home meal preparation affects energy and food group intakes among midlife women. Appetite. 2012;58(2):438–443. doi: 10.1016/j.appet.2011.12.009 22200413

[75] Choi MK , Kim TY , Yoon JS Does frequent eating out cause undesirable food choices? Association of food away from home with food consumption frequencies and obesity among Korean housewives. Ecol Food Nutr. 2011;50(3):263–280. doi: 10.1080/03670244.2011.568909 21888582

[76] Althoff T , Nilforoshan H , Hua J et al. Large-scale diet tracking data reveal disparate associations between food environment and diet. Nat Commun. 2022;13(1):267. doi: 10.1038/s41467-021-27522-y 35042849 PMC8766578

[77] Crespo-Bellido MS , Grutzmacher SK , Takata Y et al. The association between food-away-from-home frequency and a higher BMI varies by food security status in US adults. J Nutr. 2021;151(2):387–394. doi: 10.1093/jn/nxaa364 33296463

[78] European Food Safety Authority Guidance on the EU Menu methodology. EFSA J. 2014;12(12):3944. doi: 10.2903/j.efsa.2014.3944

[79] Raber M , Wolfson J The challenging task of measuring home cooking behavior. J Nutr Educ Behav. 2021;53(3): 267–269. doi: 10.1016/j.jneb.2020.11.012 33454197 PMC7954863

