# Supplementary material for: Patterns of food preparation in children and adult diets and their associations with demographic and socio-economic characteristics, health and nutritional status, physical activity, and diet quality
Source: J Nutr Sci. 2025 Jan 23;14:e10. doi: 10.1017/jns.2024.87 (PMC11811874; doi:10.1017/jns.2024.87)
Supplement: Rei et al. supplementary material [file S2048679024000879sup001.docx]

**Table S1.** Distribution of mean daily intakes (%) of foods and beverages according to the classes of food preparation considered by IAN-AF 2015/16, weighted for the distribution of the Portuguese population.

| **Mean daily intake of foods/beverages prepared:** | **% (95%CI)** |
| --- | --- |
| *At home by him/herself* | 12.2 (11.2, 13.3) |
| *At home by relatives or friends* | 18.6 (17.6, 19.5) |
| *At home, not specified* | 2.7 (2.5, 2.9) |
| *Away-from-home by food retail* (including food consumed in natura) | **45.8 (44.8, 46.9)** |
| *Away-from-home by restaurant, catering and take-away/delivery establishments* | 7.9 (7.1, 8.6) |
| *Away-from-home by school and work canteens* | 4.0 (3.5, 4.4) |
| *Away-from-home by cafeterias, snack-bars and bakeries* | 8.6 (8.2, 9.1) |
| *Away-from-home, not specified* | 0.2 (0.1, 0.4) |

**Table S2.** Distribution of the patterns of food preparation according to demographic and socioeconomic characteristics, weighted for the distribution of the Portuguese population.

|  |  | **Patterns of food preparation % (95%CI)** | | | |
| --- | --- | --- | --- | --- | --- |
|  | **n** | ***At home by themselves*** | ***At home by relatives or friends*** | ***By food retail*** (including food consumed in natura) | ***By restaurants, canteens and other away-from-home establishments*** |
| **Total** | **5005** | **16.3 (14.4, 18.2)** | **13.7 (12.0, 15.4)** | **24.1 (21.8, 26.2)** | **45.9 (43.8, 48.1)** |
| **Sex** |  |  |  |  |  |
| Women | 2613 | 24.0 (21.2, 26.8) | 7.4 (5.5, 9.3) | 27.6 (24.4, 30.8) | 41.0 (37.9, 44.1) |
| Men | 2392 | 8.3 (6.6, 9.9) | 20.4 (17.8, 22.9) | 20.2 (17.8, 22.7) | 51.1 (48.5, 53.8) |
| **Age group** |  |  |  |  |  |
| Children (3-9 years) | 521 | 0.2 (0.0, 0.6) | 7.6 (4.6, 10.7) | 23.5 (17.3, 29.7) | 68.6 (61.2, 76.0) |
| Adolescents (10-17 years) | 632 | 0.0 (0.0, 0.1) | 20.9 (16.5, 25.3) | 22.5 (17.7, 27.2) | 56.6 (50.3, 62.8) |
| Adults (18-64 years) | 3102 | 15.2 (13.1, 17.3) | 13.5 (11.4, 15.5) | 22.5 (20.2, 24.9) | 48.8 (46.0, 51.6) |
| Elderly (65-84 years) | 750 | 33.4 (27.5, 39.3) | 13.4 (9.7, 17.1) | 30.5 (25.2, 35.9) | 22.7 (18.4, 26.9) |
| **Area of residence** |  |  |  |  |  |
| Predominantly urban | 3650 | 15.6 (13.2, 18.0) | 12.6 (10.6, 14.6) | 25.0 (22.5, 27.5) | 46.8 (44.3, 49.3) |
| Medium urban | 863 | 16.9 (14.8, 19.1) | 17.5 (15.1, 19.9) | 20.1 (14.9, 25.4) | 45.4 (41.4, 49.5) |
| Predominantly rural | 492 | 21.7 (16.2, 27.2) | 17.9 (13.5, 22.2) | 21.2 (15.3, 27.2) | 39.2 (31.6, 46.8) |
| **Education*** |  |  |  |  |  |
| No education/primary | 1497 | 31.5 (26.9, 36.1) | 15.3 (12.4, 18.2) | 23.9 (20.0, 27.7) | 29.4 (25.4, 33.4) |
| Secondary | 2201 | 11.1 (9.1, 13.0) | 14.4 (11.8, 17.0) | 25.1 (22.3, 28.0) | 49.4 (46.2, 52.7) |
| Tertiary | 1291 | 7.9 (5.8, 10.0) | 10.7 (8.3, 13.1) | 21.7 (18.4, 25.0) | 59.7 (55.8, 63.6) |
| **Marital status**† |  |  |  |  |  |
| Not married | 1495 | 16.8 (13.6, 20.0) | 12.5 (9.5, 15.5) | 24.1 (20.7, 27.5) | 46.6 (42.8, 50.5) |
| Married/cohabiting | 2354 | 20.4 (17.5, 23.3) | 14.1 (12.0, 16.2) | 24.3 (20.8, 27.7) | 41.2 (38.1, 44.4) |
| **Household composition**† |  |  |  |  |  |
| Without children/adolescents | 2499 | 22.1 (19.2, 25.0) | 13.9 (11.7, 16.1) | 24.4 (21.5, 27.3) | 39.6 (36.7, 42.5) |
| With children/adolescents | 1350 | 12.9 (10.1, 15.8) | 12.6 (9.2, 16.0) | 23.8 (20.1, 27.6) | 50.6 (46.4, 54.9) |
| **Household monthly net income**† |  |  |  |  |  |
| ≤ €970 | 1377 | 29.0 (24.7, 33.2) | 15.5 (12.1, 18.9) | 22.6 (19.1, 26.2) | 32.9 (29.1, 36.7) |
| €971 – 1940 | 1389 | 15.0 (12.1, 17.9) | 13.1 (10.2, 15.9) | 26.3 (22.5, 30.1) | 45.7 (41.7, 49.6) |
| > €1940 | 708 | 7.9 (5.6, 10.2) | 10.1 (6.2, 14.0) | 24.8 (19.8, 29.8) | 57.2 (52.6, 61.8) |
| **Household food insecurity status**† |  |  |  |  |  |
| Food security | 3448 | 17.4 (15.3, 19.5) | 13.5 (11.6, 15.4) | 24.2 (21.5, 27.0) | 44.9 (42.4, 47.3) |
| Food insecurity (mild and severe) | 397 | 32.3 (26.0, 38.6) | 13.3 (8.5, 18.1) | 24.0 (18.8, 29.1) | 30.4 (24.2, 36.7) |

^*^Education completed by parents for participants <18 years; †Only participants ≥18 years.

**Table S3.** Distribution of the patterns of food preparation according to health and nutritional status, level of physical activity, and diet quality, weighted for the distribution of the Portuguese population.

|  |  | **Patterns of food preparation % (95%CI)** | | | |
| --- | --- | --- | --- | --- | --- |
|  | **n** | ***At home by themselves*** | ***At home by relatives or friends*** | ***By food retail*** (including food consumed in natura) | ***By restaurants, canteens and other away-from-home establishments*** |
| **Health status (NCDs)** |  |  |  |  |  |
| No | 3164 | 12.4 (10.4, 14.3) | 13.7 (11.6, 15.8) | 22.4 (20.2, 24.7) | 51.5 (48.7, 54.3) |
| Yes | 1841 | 22.2 (19.1, 25.4) | 13.8 (10.9, 16.6) | 26.4 (22.7, 30.2) | 37.6 (33.9, 41.2) |
| **Nutritional status (BMI)** |  |  |  |  |  |
| Normal and underweight | 2137 | 10.2 (8.3, 12.1) | 13.8 (11.3, 16.3) | 23.4 (20.6, 26.2) | 52.7 (49.5, 55.9) |
| Overweight | 1612 | 15.3 (12.4, 18.1) | 16.2 (13.0, 19.4) | 23.9 (20.4, 27.4) | 44.7 (40.6, 48.7) |
| Obesity | 1062 | 26.6 (21.8, 31.5) | 9.5 (6.4, 12.6) | 24.6 (20.7, 28.5) | 39.2 (34.9, 43.6) |
| **Level of physical activity (IPAQ)*** |  |  |  |  |  |
| Inactive | 1623 | 16.9 (14.0, 19.7) | 15.7 (12.8, 18.6) | 22.8 (19.4, 26.3) | 44.6 (40.8 ,48.4) |
| Minimally active | 1176 | 19.1 (15.7, 22.5) | 12.9 (9.7, 16.0) | 25.6 (21.6, 29.7) | 42.4 (37.5, 47.3) |
| Active | 937 | 20.7 (16.2, 25.1) | 11.6 (8.9, 14.2) | 24.3 (19.4, 29.3) | 43.4 (39.0, 47.8) |
| **Diet quality (MAI)** |  |  |  |  |  |
| Low | 1668 | 5.5 (4.0, 7.1) | 11.1 (9.0, 13.1) | 29.0 (25.3, 32.8) | 54.4 (50.8, 58.0) |
| Medium | 1668 | 14.9 (12.2, 17.6) | 14.7 (12.1, 17.4) | 20.8 (17.9, 23.6) | 49.6 (46.2, 53.0) |
| High | 1669 | 25.8 (22.1, 29.6) | 14.9 (11.7 ,18.0) | 23.1 (19.3, 26.9) | 36.2 (32.3, 40.1) |

^*^Only participants ≥18 years.

NCDs, Noncommunicable Diseases; BMI; Body Mass Index; IPAQ, International Physical Activity Questionnaire; MAI, Mediterranean Adequacy Index.
